# Vortex Lattice Instabilities in YBa_2_Cu_3_O_7-x_ Nanowires

**DOI:** 10.3390/ma11020211

**Published:** 2018-01-30

**Authors:** Víctor Rouco, Davide Massarotti, Daniela Stornaiuolo, Gian Paolo Papari, Xavier Obradors, Teresa Puig, Francesco Tafuri, Anna Palau

**Affiliations:** 1 Dipartimento di Fisica, Universitá degli Studi di Napoli Federico II, 80126 Napoli, Italy; vroucogomez@gmail.com (V.R.); stornaiuolo@fisica.unina.it (D.S.); gpaolo.papari@gmail.com (G.P.P.); tafuri@na.infn.it (F.T.); 2 Dipartimento di Ingegneria Elettrica e delle Tecnologie dell’Informazione, Università degli Studi di Napoli Federico II, 80125 Napoli, Italy; massarottidavide@gmail.com; 3 CNR-SPIN UOS Napoli, Monte Sant’Angelo, 80126 Napoli, Italy; 4 Institut de Ciència de Materials de Barcelona, CSIC, Campus de la UAB, 08193 Bellaterra, Spain; obradors@icmab.es (X.O.); teresa.puig@icmab.es (T.P.)

**Keywords:** high-temperature superconducting nanowires, flux-flow instabilities, critical vortex velocity, switching voltage jumps, single-photon detectors

## Abstract

High-resolution focused ion beam lithography has been used to fabricate YBa_2_Cu_3_O_7-x_ (YBCO) wires with nanometric lateral dimensions. In the present work, we investigate Flux-flow instabilities in nanowires of different widths, showing sudden voltage switching jumps from the superconducting to the normal state. We present an extensive study on the temperature and field dependence of the switching characteristics which reveal that voltage jumps become less abrupt as the temperature increases, and disappear at the vortex-liquid state. On the contrary, the current distribution at the critical point becomes narrower at high temperatures. Sharp voltage switchings very close to the critical current density can be obtained by reducing the width of the nanowires, making them very appealing for practical applications.

## 1. Introduction

New progress in understanding the vortex matter dynamics in high-temperature superconductors, with a rich and complex vortex phase diagram [1], is both of practical and fundamental interest. Studies performed in superconductors are predominantly focused on the mechanisms controlling vortex motion in the limit of low velocity values, very close to the critical current density, *J_c_*. However, a large number of interesting non-equilibrium physical phenomena are associated with vortex motion at high flux-flow velocities [2]. In particular, instabilities of the vortex dynamics, under certain conditions of temperature and magnetic field, induce reversible discontinuous voltage jumps, which abruptly switch the superconductor to the normal state, with very promising potential applications in ultrafast superconducting single-photon detectors [3,4,5]. In those devices, a sample is biased by a current value very close to the vortex instability, thus the incidence of a single photon produces a hot spot reducing the local critical current below the bias current with a fast switch from the superconducting to the normal state [3,6]. With the aim of increasing the reliability and capabilities of these detectors, the size of the devices should be comparable to the hot spot region (several nm) in order to switch the whole cross-section to the normal state, thus avoiding parallel superconducting currents. On the other hand, a good reproducibility and narrow distribution of voltage jumps is necessary for their optimum performance. In this direction, very extensive research has been made concerning conventional low temperature superconductors [3,4,5,6,7]. High-temperature superconductors with short coherence length and fast quasiparticle recombination appear as very promising candidates, enabling higher device operating temperatures [8,9]. However, these materials are much less explored mainly due to the challenging task of fabricating high quality High Temperature Superconductor (HTS) nanostructures. Progress in this direction, associated with recent advances in nanolithography techniques, has allowed for the reduction of the size of HTS YBa_2_Cu_3_O_7-x_ (YBCO) films down to ~50 nm, opening up the path for the design of novel HTS devices [9,10,11].

The nature of voltage jumps induced by vortex flow instabilities can be associated with different mechanisms. At temperatures close to the transition temperature, *T_c_*, the electron-phonon interaction dominates at the instability point and switching can be characterized in terms of the Larkin-Ovchinnikov (LO) instability, associated with a deviation of the quasiparticle distribution from its equilibrium, resulting in a shrinkage of the vortex core [12]. According to the LO theory, which assumes a uniform quasiparticle distribution, a magnetic field independent value of the vortex velocity at the instability point, v*, is expected [12,13]. At low magnetic fields, a non-uniform distribution of nanoparticles may be considered and a crossover field to a regime determined by a power law behavior of v* ~ *H^−^*^1/2^ is expected [14]. Both behaviors have been experimentally observed in low- and high-temperature superconductors [5,15], although other complex v*(*H*) dependences, not predicted by the LO model, have also been found at the low field regime [16]. A v* ~ *H^−^*^1/2^ power law dependence was also obtained in YBCO films measured at low temperatures, *T* << *T_c_*, where a new type of instability associated with a vortex core expansion, instead of a vortex shrink as considered in the LO model, was described [17,18]. Within all these models, however, the presence of pinning, a key parameter especially when dealing with HTS, was missing. Several experimental and theoretical works addressing this point found unconventional non-monotonic behaviors of v*(*H*) [19,20,21]. In this work, we analyze the flux-flow instabilities of the vortex lattice in high-temperature superconducting YBa_2_Cu_3_O_7-x_ (YBCO) nanowires with variable widths, offering a tantalizing potential for detector applications. Besides the attractive overall performance of superconducting nanowires as single-photon detectors [22], they are highly promising systems for future green electronics, transporting current without dissipation at nanometric scales [23], as well as for developments in topological quantum-state engineering [24,25]. In particular, superconducting quantum interference devices (SQUIDs) with high spatial resolution can be obtained, where the nanostructured superconductor acts as a weak link [26,27].

## 2. Materials and Methods

**Figure 1:**
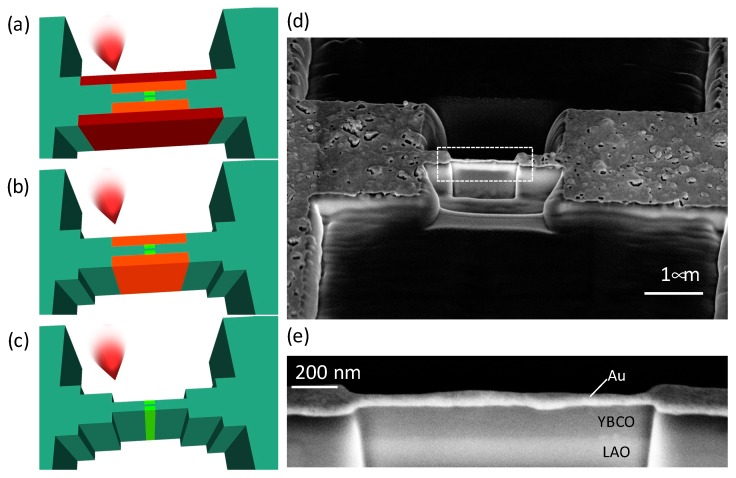
(**a**–**c**) Schematic representation of the milling process used to fabricate the nanowires. Red, orange, and yellow colors represent milling doses of 200 pA, 50 pA, and 10 pA, respectively. (**d**) Tilted SEM image of an 80-nm wide nanowire. (**e**) Zoom-in image of the nanowire cross-section (region marked with dots in (**d**)).

High quality YBCO thin films, with a thickness of 150 nm, were grown on LaAlO_3_ (LAO) single crystal substrates (CrysTec, Berlin, Germany) by chemical solution deposition (CSD). Samples were prepared from metalorganic solutions with stoichiometric quantities of Y, Ba, and Cu anhydrous trifluoroacetate (TFA), deposited by spin-coating on 5 × 5 mm^2^ substrates. After a film drying process, the organic matter of the deposited films was decomposed at ~300–400 °C and crystalized at a high temperature (~750–800 °C) in a controlled humid atmosphere. Finally, the superconducting phase was achieved by oxygen annealing at 450 °C. Sample texture was characterized by full X-ray diffraction *θ*–2*θ* spectrums showing a good epitaxial growth, with just (*00l*) reflections detected [28]. We initially prepared, by standard optical photo-lithography, YBCO micro-bridges with a width of 30 µm and a length of 100 µm, in a four-point configuration. Patterned bridges were covered with a protecting Au capping layer of 50 nm, grown by thermal evaporation, to avoid oxygen desorption or sample degradation during the nanowire fabrication [29,30]. The width of the initial micro-bridge was reduced to the nanoscale by performing lateral cuts using a Carl Zeiss Crossbeam 1560 XB system (Carl Zeiss AG, Oberkochen, Germany) equipped with a scanning electron microscope (SEM) and a focused ion beam (FIB) column. This very powerful technique can be used to direct fabricate complex 3D nanometric structures by FIB lithography while imaging the sample by SEM. An accurate optimization of the FIB milling parameters (beam voltage, beam current, and dose) was performed in order to obtain high-resolution nanoscale patterns without damaging the superconducting properties during the milling process [31,32]. The patterned micro-bride was narrowed by performing several steps with successive cuts, in which we decreased the current dose from 200 pA to 10 pA, using a constant ion energy of 30 keV, as sketched in Figure 1a–c. A cross-section SEM image of a patterned nanowire is shown in Figure 1d, where the different cuts can be seen. A zoomed-in view corresponding to the white rectangle is displayed in Figure 1e, where a good homogeneity of the YBCO patterned nanowire through the thickness can be clearly appreciated.

**Figure 2:**
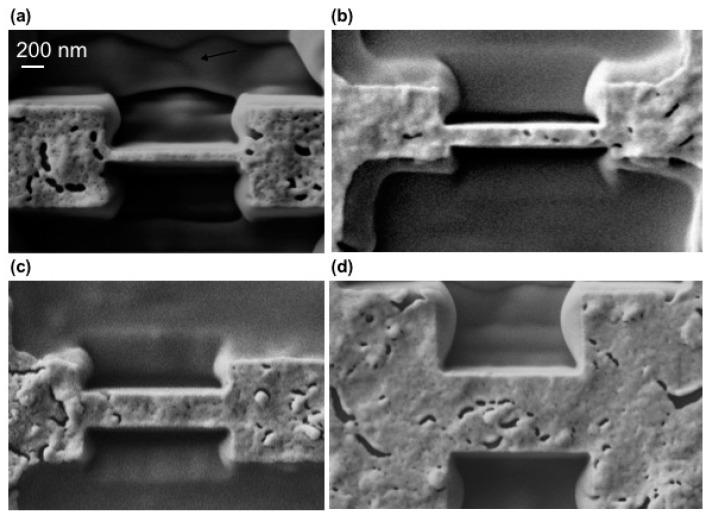
SEM images of patterned nanowires width (**a**) *w* = 80 nm, (**b**) *w* = 250 nm, (**c**) *w* = 300 nm, and (**d**) *w* = 700 nm.

By using this approach, we were able to fabricate nanowires ~1 µm in length with the desired width, *w*, from *w* = 700 nm down to *w* = 80 nm, maintaining good superconducting performances. Figure 2 shows top view SEM images of the different nanowires used for this study. The critical temperature of patterned nanowires was in the range of *T_c_* ~ 87–92 K, and the critical current density was *J_c_* ~ 3–5 MA/cm^2^ at 77 K and in the self-field. It should be noted that before measuring the nanowires we ensured that they exhibited a homogeneous cross-section, without any porosity, precipitates, or other visible defect (as observed in Figure 1e) in order to obtain reliable results. The porosity and defects observed in the top view images shown in Figure 2 correspond to the Au capping layer that was deposited on top of the YBCO to prevent any damage during the FIB milling process.

In-field electrical transport measurements were performed in a 9 T Physical Property Measurement System from Quantum Design (San Diego, CA, USA). We measured current-voltage curves (*I*-*V*) at different temperatures and magnetic fields, up to the switching point. The applied magnetic field was kept perpendicular to the film and the applied current was maintained in maximum Lorentz Force configuration. Additionally, self-field transport measurements and switching current distributions were realized in a Heliox VL Oxford Instruments system (Oxford Instruments, Oxford, UK) equipped with homemade lines and EMI, RC, and Cooper Powder filters. Switching current distributions at different temperatures were performed by current biasing the nanowires with a ramp at a constant rate of 155 mA/s, using a threshold voltage of 50 mV. The bias current at the critical point was recorded by repeating the measurement 5000 times, allowing us to obtain switching current distribution histograms [33,34].

## 3. Result and Discussion

**Figure 3:**
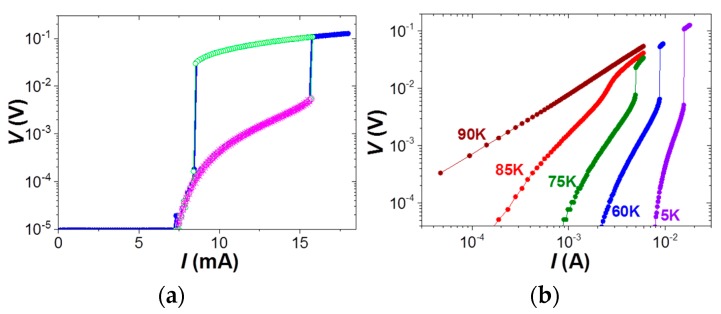
(**a**) *I-V* characteristics measured at 5 K and 1 T on a 300-nm-wide nanowire for a maximum applied current *I_max_* = 18 mA (closed blue circles), 16 mA (open green circles), and 15.8 mA (magenta stars). (**b**) *I*-*V* curves measured at 1 T for a 300-nm-wide nanowire at different temperatures.

Figure 3a displays the current-voltage, *I*-*V* characteristics measured for a 300-nm-wide nanowire at 5 K and 1 T. *I*-*V* curves were obtained by increasing the current up to a maximum value, *I_max_*, and decreasing it back to zero. It is worth noting that an identical behavior is obtained for the two curves measured at *I_max_ =* 16 mA and 18 mA. A sharp voltage transition from the superconducting to the normal state is produced at the critical point, determined by the critical current and voltage values I* = 15.8 mA and V* = 5 mV, respectively. The superconducting state is recovered by decreasing the current to zero again. As we will show below, repeated cycling of the current pulses gives identical results, signifying good reproducibility of the sharp voltage transition induced. However, a complete reversible curve is found if we apply a maximum current value just below the critical point, *I_max_* = 15.7 mA < I*, suggesting that an important role of the switching mechanism is played by flux-flow instabilities rather than thermal self-heating effects. A set of *I*-*V* curves measured by increasing the applied current, at 1 T and different temperatures, is shown in Figure 3b. A very sharp transition is observed in the curves measured at 75 K, 60 K, and 5 K, with a larger voltage jump to the normal state obtained at lower temperatures. The switching becomes much softer as the temperature is increased closer to *T_c_*.

**Figure 4:**
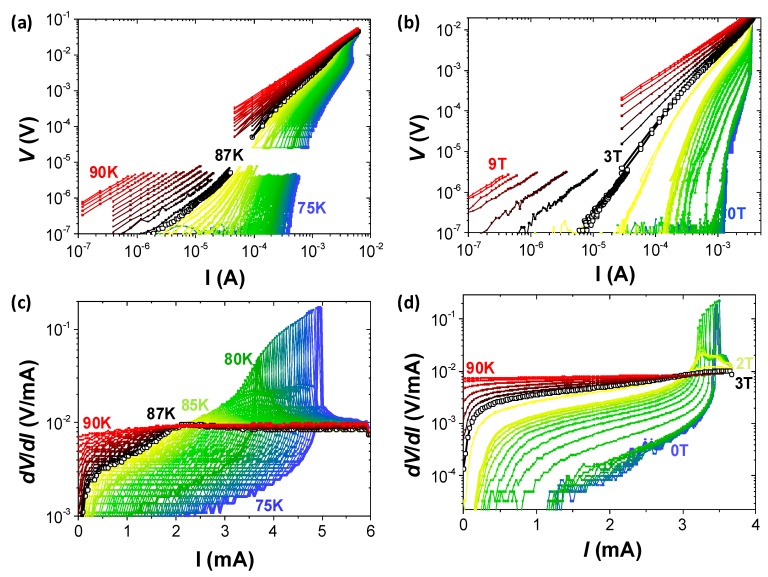
(**a**) *I*-*V* characteristics measured for a 300-nm-wide nanowire at (**a**) 1 T and different temperatures ranging from 75 K to 90 K with steps of 0.2 K. (**b**) 82 K at different applied magnetic fields ranging from 0 to 9 T with steps of 10 mT. (**c**,**d**) Derivative values *dV*/*dI* of curves shown in (**a**,**c**), respectively. Open symbols show the irreversibility points.

Figure 4 depicts a complete picture of the switching process at different temperatures and magnetic fields. Figure 4a shows the *I*-*V* characteristics obtained for a 300-nm-wide nanowire at 1 T and for a continuous range of temperatures from 75 K to 90 K. Curves measured at low temperatures show a negative curvature, characteristic of a vortex-solid state. The transition to the vortex-liquid state is determined at the irreversibility temperature, *T_irr_*, where an inversion of the curvature is detected [35] (curve depicted with open symbols at *T_irr_* = 87 K in this case). The associated resistance values, determined at each point of the *I*-*V* curves as *dV/dI*, are shown in Figure 4c. This kind of representation is very convenient to identify the curves that undergo a voltage switching transition, since at the switching point the value of *dV*/*dI* becomes higher than the resistance in the normal state.

We evaluate the value of the normal state resistance from the *I*-*V* curve measured at 90 K, being ~0.01 V/mA, and we observe that vortex instabilities show lower voltage jumps as the temperature increases, and completely disappear at the vortex-liquid state. Figure 4b,d show the same analysis performed at a fixed temperature of *T* = 82 K as a function of the magnetic field, showing similar features, with vortex instability jumps observed below the irreversibility field of *H_irr_* ~ 3 T.

**Figure 5:**
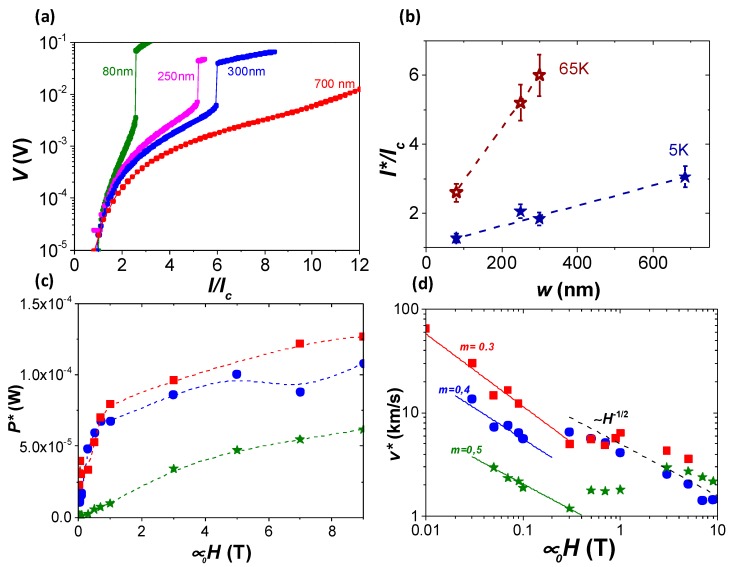
(**a**) *I*-*V* characteristics obtained for different nanowires at 65 K and 1 T. (**b**) I*/I_c_ as a function of the width of the nanowire, at 1 T and different temperatures. (**c**) Magnetic field dependence of P* at 5 K, for nanowires with *w* = 700 nm (squares), 300 nm (circles), and 80 nm (stars). (**d**) Magnetic field dependence of v* at 5 K, for nanowires with *w* = 700 nm (squares), 300 nm (circles), and 80 nm (stars). Dashed lines indicate a v* ~ H^−^^1/2^ and solid lines v* ~ H^−m^ dependences.

In the following, we show the analysis of vortex instabilities performed in nanowires of different widths. Figure 5a depicts the *I*-*V* characteristics obtained at 65 K and 1 T, normalized to its self-field current density, *I_c_*. Abrupt voltage jumps are observed in all cases at different values of I* and V*. Figure 5b plots the value of the current at the switching point, I*, normalized to *I_c_*, determined at 1 T and two different temperatures, as a function of the nanowire width, *w*. We found that the switching current can be finely tuned by means of the applied magnetic field, temperature, and through the modification of the nanowire width. A monotonic behavior is observed with a decrease of the ratio I*/I_c_ approaching the value of I*/I_c_ ~ 1, as reducing *w*. This tendency reveals the high potentiality to use narrow nanowires in detector devices based on temperature-induced voltage switching jumps. Narrow nanowires with values of I*/I_c_ ~ 1 would be operative by using current bias values below *I_c_*, thus strongly reducing dissipative heating effects.

With the aim of analyzing the nature of the vortex instability jumps in the patterned nanowires, we evaluated the dissipation power, P*, and dissipation vortex velocity, v*, at the critical point. From the non-hysteretic curve shown in Figure 3a, self-heating effects appear to be excluded as the main mechanism in the switching process. Further evidence of that is given in Figure 5c, which shows the dissipation power, evaluated as P* = I*V*, for different nanowires at 5 K, as a function of the magnetic field. A monotonic increasing dependence is found, which dismisses thermal runaway mechanisms as being mainly responsible for the voltage jumps, since in that case P* should be magnetic field-independent [36]. The vortex velocity at the critical point was determined as v* = V*/Lµ_0_*H*, where *L* is the length of the nanowire. Figure 5d shows the values of v* obtained for several nanowires at 5 K as a function of the magnetic field. A clear v*(*H*) dependence is observed for the whole magnetic field range evaluated, which is not consistent with the constant v* values predicted by the LO instability theory [12,13]. At high fields (*H* > 1 T), the values of v* obtained for the different nanowires merge, following a power low dependence that can be fitted as v* ~ H^−^^1/2^, also observed in many other systems, and associated to different mechanisms [14,18,19]. At low fields, the v* values differ from sample to sample, being lower for the narrower nanowires. In this regime, the magnetic field dependence of v* can be fitted with a power law behavior with a variable exponent, v* ~ H^−m^, in agreement with vortex instability processes affected by pinning effects [22]. Although the specifics of the origin of the observed power law dependencies are beyond the scope of this work, the role of mesoscopic effects and vortex lattice rearrangements, essential when dealing with nanometric bridges [31,37], should also play a key role.

**Figure 6:**
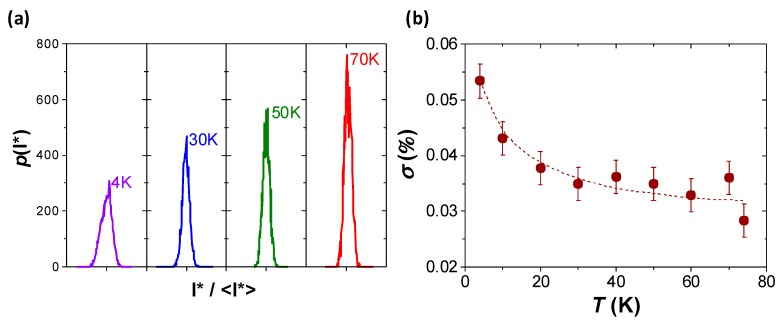
(**a**) Histograms of probability density of the switching current for a 300-nm-wide nanowire, p(I*), as a function of I*, normalized to the average value <I*>. The X-axis extends from I*/<I*> = 0.996 to 1.004 in all of the histograms measured at different temperatures. (**b**) Temperature dependence of the standard deviation obtained from the *p*(I*) histograms.

Finally, we discuss on the reproducibility of the switching process in YBCO nanowires. To do so, we measured a large number of *I-V* curves at an applied field of zero and a fixed temperature for a nanowire with *w* = 300 nm, and we determined the number of curves that switched at a certain I* value. Figure 6a shows the obtained histograms with the probability density of the switching current, p(I*), centered at the midpoint <I*>, for different temperatures. We obtained very narrow switching current distributions with some small broadening as the temperature is lowered. Figure 6b shows the temperature evolution of the standard deviation, σ, obtained from several histograms. A very good reproducibility of the switching point is observed at all temperatures, with σ *~* 0.03% at high temperatures (T > 20 K) and a small monotonic increase up to σ *~* 0.05% at 4 K. This result indicates that by tuning the operating temperature one can tip the balance among the height of the voltage jump and the width of the switching current distribution. By increasing the temperature, very narrow distributions can be obtained, although in this case the voltage jumps will be less pronounced (Figure 3b). Although the analysis of the switching current distribution was performed on a particular nanowire, we show that the switching critical point can be finely tuned by the nanowire width (Figure 5b), pointing out that the reproducibility of the instability jumps if a homogenous nanowire cross-section is assured (as depicted in Figure 1e).

## 4. Conclusions

Flux-flow instabilities were studied in YBCO nanowires patterned by high-resolution focused ion beam lithography. Patterning conditions were fully optimized in order to achieve high quality superconducting nanowires of different widths, ranging from 700 nm to 80 nm. Sharp switching transitions from the superconducting to the normal state were observed in *I*-*V* characteristics for all nanowires, over a large range of magnetic fields and temperatures, in the vortex-solid state. The vortex velocity at the critical point appears to be dependent on the nanowire width, showing complex magnetic field dependences, indicating that pinning, vortex rearrangement, and mesoscopic effects may play a crucial role in the nature of vortex instability jumps. Very reproducible abrupt voltage jumps with narrow switching current distributions were obtained where the switching point can be tuned by choosing the operating temperature, magnetic field, and nanowire width. In nanowires with widths of 50 nm, the switching current at the critical point approaches the critical current density, being particularly interesting for practical applications.
